# Association of oxidative stress, inflammation, and ADIPOQ gene expression in young adults at risk of non-alcoholic fatty liver disease and cardiovascular disease

**DOI:** 10.11604/pamj.2026.53.175.47681

**Published:** 2026-04-27

**Authors:** Rachana Raveendran, Anthony Josephine, Varalakshmi Sureka, Periandavan Kalaiselvi, Poornima Raja Raja Varma, Lasitha Neroth Kandy, Renjith Kariyil Radhakrishnan Nair, Manjusha Kottola, Midhun Thazhissery Mohanan, Swathi Thoduvayil, Thahira Abdulla, Pinchulatha Kottiyath, Dinesh Roy Divakaran

**Affiliations:** 1 Meenakshi Academy of Higher Education and Research (Deemed to be University), Chennai, Tamil Nadu, India,; 2 Department of Research, Meenakshi Academy of Higher Education and Research (Deemed to be University), Chennai, Tamil Nadu, India,; 3 Department of Medical Biochemistry, Dr. A.L.M. PG Institute of Basic Medical Sciences, University of Madras, Tamil Nadu, India,; 4 Genetika, Centre for Advanced Genetic Studies, Thiruvananthapuram, Kerala, India

**Keywords:** ADIPOQ gene expression, cardiovascular disease, inflammatory markers, non-alcoholic fatty liver disease, oxidative stress

## Abstract

**Introduction:**

non-alcoholic fatty liver disease (NAFLD) and cardiovascular diseases (CVD) are increasingly seen in young adults due to obesity, sedentary lifestyle, and poor diet. Oxidative stress and chronic inflammation are key contributors to their development, promoting liver damage, endothelial dysfunction, and atherosclerosis. Adiponectin, encoded by the ADIPOQ gene, has protective metabolic effects, and its reduction, often influenced by genetic factors, is linked to increased disease risk. This study evaluates the relationship between oxidative stress (MDA), inflammation (IL-6), and ADIPOQ gene expression in young adults to identify early biomarkers.

**Methods:**

this case-control study (2022 December-2024 December) included 300 participants aged 18-45 years with clinically established non-alcoholic fatty liver and/or cardiovascular risk disease, who were categorized as the case group. The remaining 150 healthy individuals served as the control group. MDA and IL-6 levels were measured using ELISA, while ADIPOQ expression was analyzed via reverse transcription polymerase chain reaction (RT-PCR). Data were analyzed using t-tests, receiver operating characteristic (ROC) analysis, and quantile regression in Stata 17.0, with significance set at p<0.05.

**Results:**

cases showed elevated MDA and IL-6 levels and reduced ADIPOQ expression compared to controls. ROC analysis identified MDA as the strongest biomarker (AUC = 0.9605). IL-6 showed diagnostic potential but lost significance after adjustment for sociodemographic variables. MDA and IL-6 levels negatively correlated with ADIPOQ expression.

**Conclusion:**

oxidative stress and inflammation are associated with reduced ADIPOQ expression in young adults at risk of NAFLD and CVD. While ADIPOQ alone may have limited predictive power, early identification of oxidative and inflammatory markers could aid in timely intervention.

## Introduction

Non-alcoholic fatty liver disease (NAFLD) and cardiovascular diseases (CVD) have emerged as significant public health challenges, now affecting younger populations who were previously considered at low risk for such metabolic disorders. The global incidence of NAFLD among adolescents and young adults has risen markedly, primarily driven by lifestyle shifts characterized by unhealthy dietary patterns, sedentary behavior, and escalating rates of obesity [1]. Alarmingly, both NAFLD and CVD often develop insidiously, advancing to severe liver pathology or cardiovascular complications in the absence of overt clinical symptoms. This silent progression underscores the need to identify early biomolecular indicators to facilitate timely detection, risk stratification, and preventive intervention.

Oxidative stress and chronic low-grade inflammation are increasingly recognized as central drivers in the pathogenesis of non-alcoholic fatty liver disease (NAFLD) and cardiovascular disease (CVD). The imbalance between reactive oxygen species (ROS) production and antioxidant defenses leads to cellular damage, lipid peroxidation, and mitochondrial dysfunction, exacerbating disease progression [2]. In the context of NAFLD, excessive ROS leads to lipid peroxidation, damaging hepatocytes and activating hepatic stellate cells, which drives fibrogenesis. This oxidative injury accelerates the progression from simple steatosis to non-alcoholic steatohepatitis (NASH), heightening the risks of fibrosis and cirrhosis. Targeting oxidative stress with antioxidant therapies and dietary interventions shows promising potential for NAFLD management [3]. In parallel, oxidative stress plays a crucial role in the development of cardiovascular disease by impairing endothelial function, reducing nitric oxide availability, and triggering vascular inflammation. Reactive oxygen species (ROS) contribute to the oxidation of low-density lipoproteins (LDL), facilitating the formation of foam cells and the buildup of atherosclerotic plaques. This sequence of events promotes the advancement of atherosclerosis and dramatically elevates the likelihood of cardiovascular events [4]. Chronic inflammation further exacerbates these conditions. Elevated levels of pro-inflammatory cytokines, such as interleukin-6 (IL-6), are commonly observed in individuals with NAFLD and CVD. These cytokines sustain hepatic and vascular inflammation and disrupt insulin signaling pathways, thereby contributing to insulin resistance, a pivotal mechanism linking NAFLD and CVD [5].

Adiponectin, an adipose-derived hormone encoded by the ADIPOQ gene, plays a key role in metabolic regulation through its anti-inflammatory, insulin-sensitizing, and cardio-protective effects. Its levels decline with increasing adiposity, and this reduction is linked to heightened oxidative stress and inflammation, both central to the development of NAFLD and CVD [6]. Beyond environmental factors, genetic variations play a crucial role in regulating adiponectin expression and activity. Specific polymorphisms in the ADIPOQ gene, such as rs266729 (-11377C/G) and rs2241766 (+45T/G), have been associated with reduced adiponectin levels and a heightened risk of metabolic conditions like non-alcoholic fatty liver disease (NAFLD) and coronary artery disease (CAD). These genetic alterations may influence gene expression or alter adiponectin´s bioactivity, contributing to individual susceptibility to metabolic dysfunction. Emerging research highlights the potential of these variants not only as biomarkers for early metabolic risk detection and as therapeutic targets to counteract the harmful effects of obesity-driven oxidative stress and inflammation [7].

Given adiponectin´s pivotal role in regulating insulin sensitivity, inflammation, and oxidative balance, the expression of the ADIPOQ gene holds promise as an early molecular marker for identifying metabolic dysfunction. Evaluating ADIPOQ gene expression alongside oxidative stress (MDA) and inflammatory (IL-6) markers provides a comprehensive framework to elucidate the molecular mechanisms driving NAFLD and CVD in young individuals at risk. Such an integrative approach could facilitate the development of targeted, early interventions to reduce long-term cardiometabolic complications.

Despite the growing global evidence connecting adiponectin, oxidative stress, and inflammation with early metabolic disturbances, integrative research in the Indian population, particularly among young adults, remains scarce. This demographic, increasingly exposed to lifestyle-related metabolic risks, is underrepresented in molecular studies. To date, no Indian investigation has comprehensively explored the interplay between oxidative stress, inflammatory mediators, and ADIPOQ gene expression in young adults predisposed to cardiometabolic disorders.

Given adiponectin´s integral role in mediating insulin sensitivity, inflammation, and oxidative balance, the expression level of the ADIPOQ gene may serve as a critical molecular marker for the early identification of metabolic dysfunction. Investigating ADIPOQ gene expression, particularly in conjunction with markers of oxidative stress (MDA) and inflammation (IL-6), offers a comprehensive approach to understanding the molecular underpinnings of NAFLD and CVD in young at-risk individuals. This integrated assessment may aid in the development of early, personalized intervention strategies aimed at mitigating long-term cardiometabolic risk.

## Methods

**Study design:** this was a case-control study conducted between December 2022 and December 2024 in accordance with the Declaration of Helsinki (1964). The study received ethical approval from the Institutional Ethics Committee of Genetika (Ref No: 09/2022/IECG). All participants provided informed consent prior to enrollment.

**Setting:** participants were recruited from Hridayalaya Clinic, Lords Hospital, the World Heart Day Medical Camp, and via referrals from multiple blood collection centres in Thiruvananthapuram, Kerala, India. Laboratory and genetic analyses were carried out at Genetika, Centre for Advanced Genetic Studies.

**Participants:** a total of 300 individuals aged 18 to 45 years were enrolled. The case group (n = 150) included participants with clinically diagnosed non-alcoholic fatty liver and/or cardiovascular risk disease. The control group (n = 150) comprised age- and sex-matched healthy individuals without any diagnosed chronic conditions. To reduce selection bias, both cases and controls were recruited from the same geographic and healthcare settings and matched by age and sex. Measurement bias was minimized by following standardized protocols for biochemical and genetic assays, and all analyses were performed blinded to case-control status.

**Inclusion and exclusion criteria:** inclusion criteria were individuals aged 18-45 years with a confirmed diagnosis of non-alcoholic fatty liver disease and/or cardiovascular risk factors. Exclusion criteria included individuals outside the age range, with chronic illnesses, malignancies, under prolonged pharmacological treatment, or with prior exposure to ionizing radiation, chemotherapy, or other mutagenic agents.

**Variables:** the primary outcome variable was ADIPOQ gene expression. Secondary outcome variables included biochemical markers such as malondialdehyde (MDA) and interleukin-6 (IL-6). Other variables assessed were demographic and clinical characteristics, including age, sex, body mass index (BMI), and clinical diagnosis.

**Data sources and measurement:** structured questionnaires were used to collect demographic, lifestyle, and clinical data. Venous blood (6-8 mL) was collected under fasting conditions into plain and EDTA-coated tubes. ELISA kits for MDA and IL-6 were purchased from Origin Diagnostics (catalog nos. OPK8428 and OPK1156, respectively), and the assays were validated according to the manufacturer´s instructions and previously published protocols. For gene expression, total ribonucleic acid (RNA) was isolated using a commercial extraction kit and quantified via BIO-spectrophotometry. Complementary DNA (cDNA) synthesis was performed using 50 μg of total RNA and reverse transcriptase. Real-time PCR was conducted using gene-specific primers for ADIPOQ: forward: 5´-CAGGCCGTGATGGCAGAGATG-3´; reverse: 5´-GGTTTCACCGATGTCTCCCTTAG-3´.

Quantitative real-time PCR (qRT-PCR) was carried out using gene-specific primers for ADIPOQ on a CFX Opus 96 Real-Time PCR System (Bio-Rad, USA). PCR reactions were prepared using SYBR Green Master Mix (Bio-Rad, Lot No.: 103024 RMM), and relative expression was calculated using the 2^-ΔΔCT method with GAPDH as the internal control as described by Schmittgen *et al*. 2008 [8].

**Bias:** to reduce selection bias, both cases and controls were recruited from the same geographic and healthcare settings and matched by age and sex. Measurement bias was minimized by following standardized protocols for biochemical and genetic assays, and all analyses were performed blinded to case-control status.

**Study size:** sample size was calculated using the formula:


$$n=\frac{Z^{2}pq}{d^{2}}$$

Where Z is the standard normal deviate, p is the expected prevalence derived from prior studies, q is 1 - p, and d is the allowable error margin. Prevalence estimates from previously published research were used to justify the enrollment of 150 cases and 150 controls [9]. The final sample size was adjusted for feasibility and equal group allocation in the case-control design. Assuming prevalence (p) = 25% (0.25); q = 1 - p = 0.75; Z = 1.96 (95% confidence); allowable error (d) = 5% (0.05):


$$n=\frac{{\left(1.96\right)}^{2}\times 0.25\times 0.75}{{\left(0.05\right)}^{2}}$$

$$n=\frac{0.7203}{0.0025}=288.12$$

**Quantitative variables:** quantitative variables, including gene expression levels, MDA, and IL-6 concentrations, were recorded as continuous variables. Age and BMI were also treated as continuous variables, while clinical status (case/control) was categorical.

**Statistical methods:** descriptive statistics, including means, standard deviations, and frequency distributions, were computed. Group comparisons were made using independent t-tests for normally distributed variables. ROC curve analysis was used to assess diagnostic performance, reporting area under the curve (AUC), sensitivity, and specificity. Quantile regression analysis was performed to explore the relationship between ADIPOQ gene expression (measured using the 2^-ΔΔCT method) and clinical variables. Firth logistic regression was employed to evaluate the association between biomarkers and disease status, particularly due to its robustness in handling small sample sizes and rare events, which can lead to biased estimates in standard logistic regression. Levene´s test was applied to assess the equality of variances between groups prior to conducting parametric comparisons, ensuring the assumption of homoscedasticity was met. In instances where traditional assumptions may be violated or sample distributions appear non-normal, bootstrap methods were used to compute more reliable p-values, enhancing the robustness of the inference drawn from the data. All analyses were conducted using Stata 17.0, with a significance level set at p < 0.05.

**Ethical approval:** ethics approval (09/2022/IECG) was secured from the Institutional Ethics Committee of Genetika.

## Results

**Participants:** a total of 300 young adults aged between 18 and 45 years were included in the study. The participants were divided into two groups: a case group comprising 150 individuals clinically diagnosed with non-alcoholic fatty liver disease (NAFLD) and/or exhibiting cardiovascular risk factors, and a control group of 150 age- and sex-matched healthy individuals without NAFLD or cardiovascular risk. All participants underwent complete clinical evaluation and laboratory testing.

**Descriptive data:** baseline clinical and biochemical characteristics of the study population are presented in Table 1. The analysis revealed that malondialdehyde (MDA) levels were significantly higher in cases compared to controls, with a P-value of less than 0.01, indicating elevated oxidative stress among individuals with NAFLD and cardiovascular risk. Similarly, interleukin-6 (IL-6) levels were markedly increased in the case group (P < 0.01), pointing to enhanced systemic inflammation. In contrast, the expression levels of the adiponectin-encoding gene (ADIPOQ) were significantly lower in the case group (P < 0.01), suggesting a diminished metabolic protective effect mediated by adiponectin in these individuals.

**Table 1:** comparative analysis of clinical parameters between non-alcoholic fatty liver and/or cardiovascular risk disease (cases) and healthy individual controls, based on Levene’s test for equality of variances and bootstrap P-values

Variable name	Levene's test P-value	Bootstrap P-value
Malondialdehyde (MDA) (nmol/mL)	P<0.01	P<0.01
Interleukin 6 (IL 6 ) (pg/mL)	0.802	P<0.01
Adiponectin-encoding gene (ADIPOQ) (2^-ΔΔCT)	P<0.01	P<0.01

**Figure 1:**
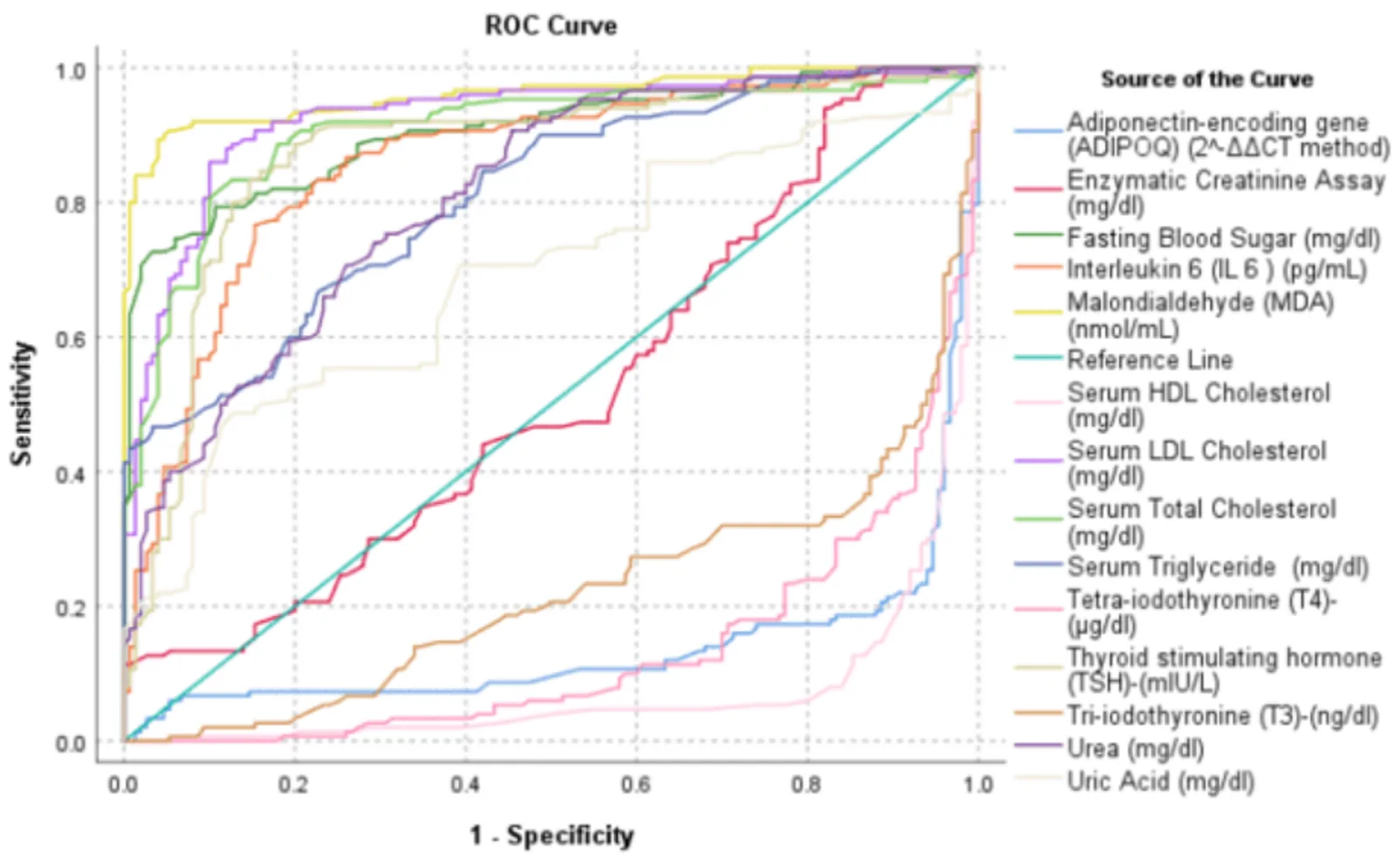
receiver operating characteristic (ROC) curves showing the diagnostic performance of clinical and biochemical markers in differentiating non-alcoholic fatty liver and/or cardiovascular risk disease (cases) from healthy controls

**Outcome data:** the discriminative ability of each biomarker to differentiate between cases and controls was assessed using receiver operating characteristic (ROC) analysis. The results, illustrated in Figure 1, showed that MDA exhibited the highest predictive accuracy, with an area under the curve (AUC) of 0.9605, reflecting excellent discriminative power. IL-6 also demonstrated strong predictive capacity, with an AUC exceeding 0.85, underscoring its role in inflammatory processes associated with disease status. On the other hand, ADIPOQ gene expression yielded an AUC close to 0.5, indicating poor predictive performance and limited utility as a standalone biomarker for classification within this population.

**Main results:** to further evaluate the predictive role of IL-6, Firth logistic regression analysis was performed, which is particularly suited for datasets with small sample sizes or sparse events. In the initial model (model 1), which included only key clinical variables, IL-6 did not emerge as a statistically significant predictor of case status (odds ratio = 0.958, 95% confidence interval: 0.794-1.156, p > 0.05). This lack of statistical significance persisted even after adjusting for sociodemographic variables in model 2 (odds ratio = 0.948, 95% confidence interval: 0.744-1.208, p > 0.05). These findings suggest that, although IL-6 levels were elevated in affected individuals, the cytokine alone does not independently predict disease presence when other factors are considered.

**Figure 2:**
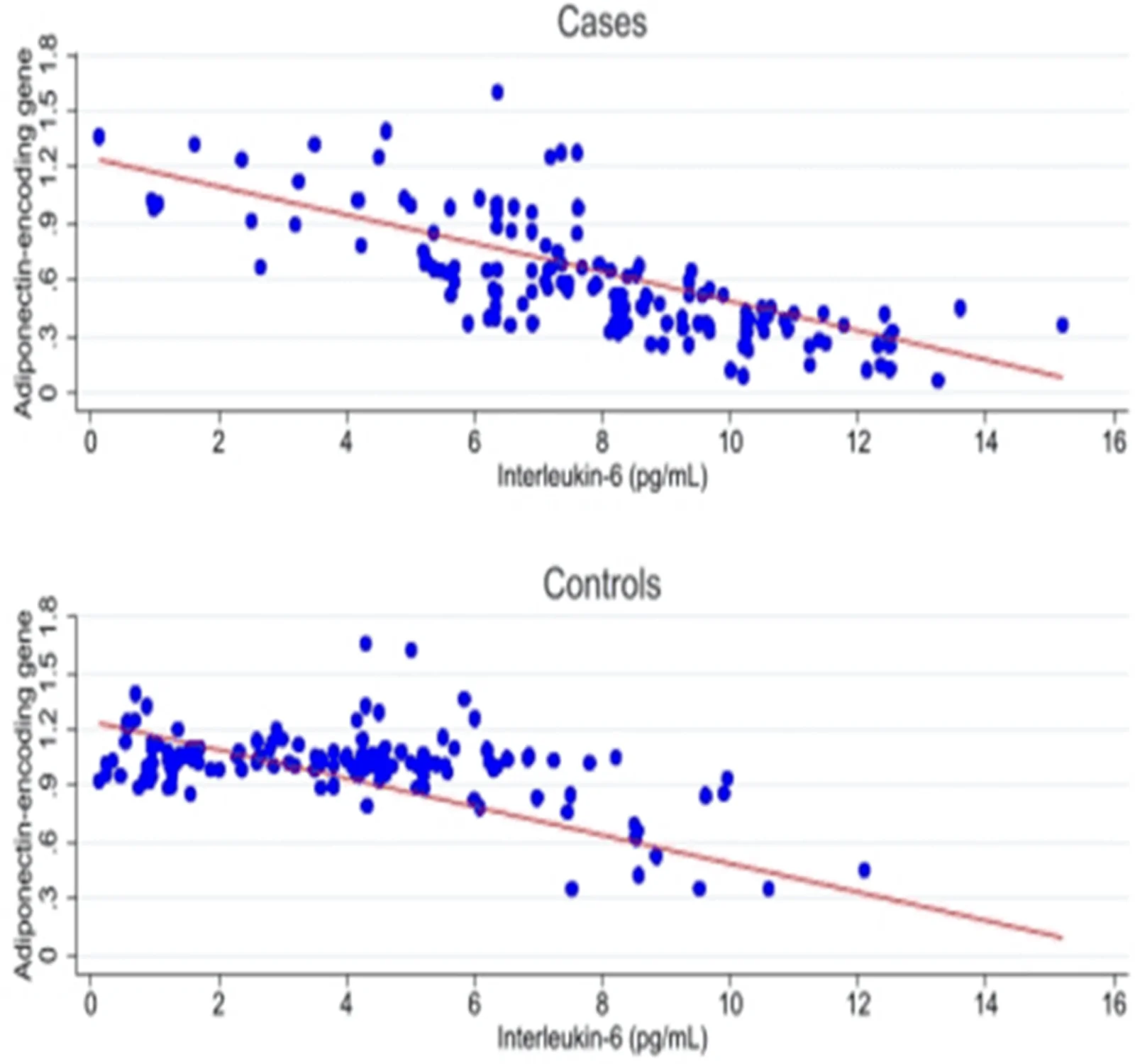
scatter plots showing the inverse relationship between Interleukin-6 (IL-6) levels and adiponectin-encoding gene (ADIPOQ) expression among cases (individuals with non-alcoholic fatty liver and/or cardiovascular risk disease) and healthy controls

**Other analyses:** further analyses examined the relationship between inflammatory and oxidative markers and ADIPOQ gene expression. A negative correlation was observed between IL-6 levels and ADIPOQ expression in both the case and control groups. This inverse relationship was more pronounced in the case group, where higher IL-6 concentrations were associated with a marked reduction in ADIPOQ expression, and the data showed wider variability, indicating considerable metabolic heterogeneity in individuals with NAFLD and cardiovascular risk. Control individuals also exhibited a negative trend, though with tighter clustering of data points, implying a more stable regulatory mechanism in the absence of systemic inflammation, as depicted in Figure 2.

**Figure 3:**
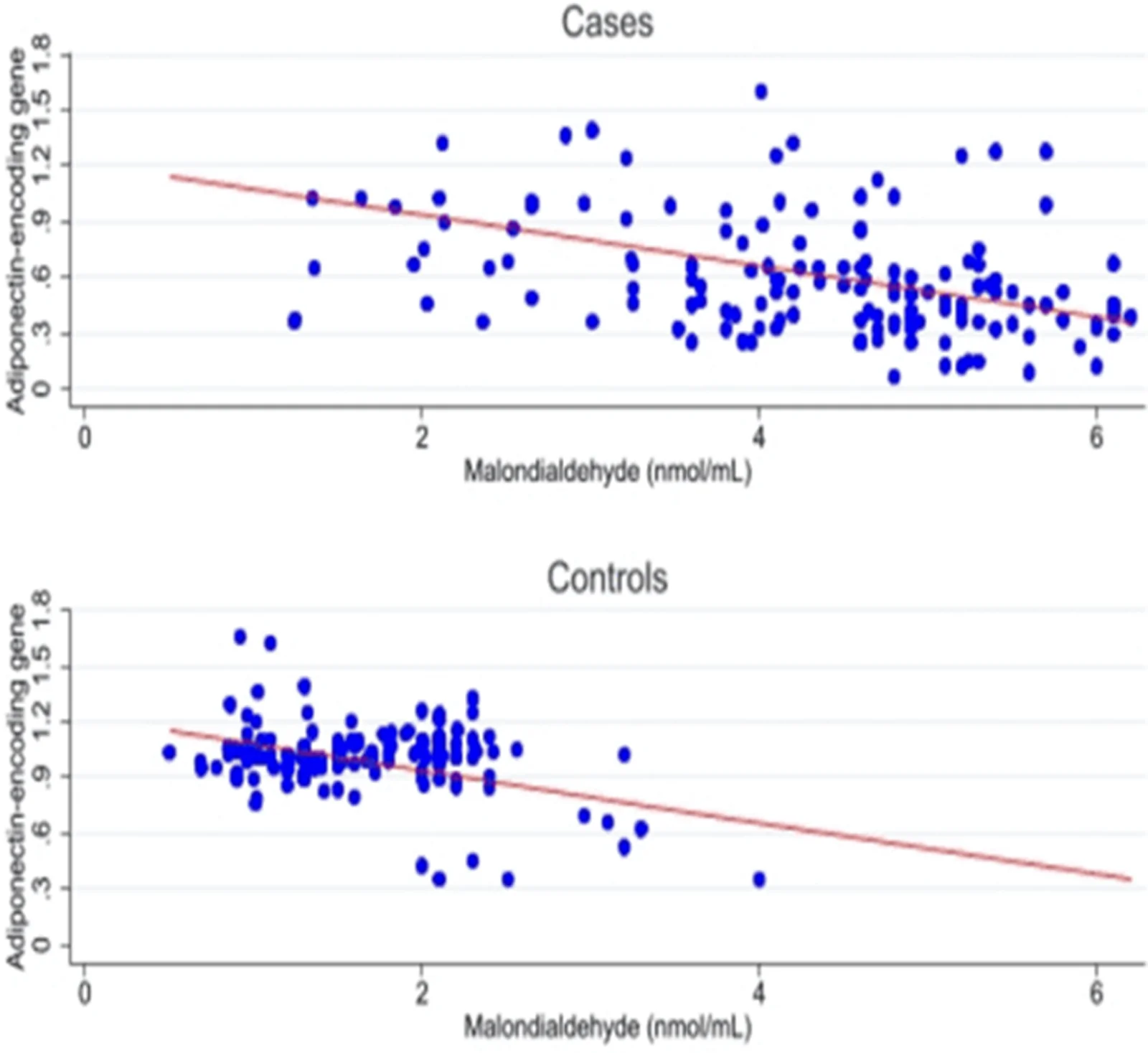
scatter plots depicting the relationship between malondialdehyde (MDA) levels and adiponectin-encoding gene (ADIPOQ) expression among cases (individuals with non-alcoholic fatty liver and/or cardiovascular risk disease) and healthy controls

Similarly, a negative correlation was identified between MDA levels and ADIPOQ expression in both groups. Among cases, as oxidative stress increased, ADIPOQ expression declined, further underscoring the impact of oxidative damage on adiponectin-mediated pathways. While this inverse association was also present in controls, the effect was less pronounced, with a narrower distribution of MDA values, as shown in Figure 3. These patterns support the hypothesis that both inflammation and oxidative stress may play key roles in suppressing adiponectin gene expression, particularly in individuals with underlying metabolic disturbances.

## Discussion

**Main findings:** the study found that young adults with NAFLD and/or cardiovascular risk had significantly higher MDA and IL-6 levels and lower ADIPOQ gene expression, indicating increased oxidative stress, inflammation, and reduced metabolic protection. MDA showed the highest predictive accuracy (AUC = 0.9605), while ADIPOQ had poor diagnostic value. IL-6 was not independently associated with disease status after adjustments. Negative correlations were observed between IL-6, ADIPOQ, and MDA and ADIPOQ, especially in cases suggesting inflammation and oxidative stress may suppress adiponectin expression in disease states.

**Novelty of the study:** this study uniquely investigates the biomolecular landscape of young adults aged 18-45 years, a relatively underrepresented group in the context of non-alcoholic fatty liver disease (NAFLD) and cardiovascular risk, by integrating oxidative stress (MDA), systemic inflammation (IL-6), and gene-level expression of ADIPOQ. While earlier studies have focused mainly on middle-aged or elderly populations, emerging evidence suggests that metabolic and cardiovascular alterations begin much earlier in life [10,11]. However, limited data are available that simultaneously examine inflammatory, oxidative, and adipokine gene expression signatures in younger adults, making this study especially relevant in early risk detection and prevention.

Previous studies have identified MDA and IL-6 as reliable oxidative stress and inflammation markers in NAFLD and cardiovascular diseases [12,13]. Similarly, low adiponectin levels have been associated with insulin resistance and atherosclerosis [14,15]. However, few studies have examined ADIPOQ gene expression specifically, particularly its correlation with MDA and IL-6 within the same cohort. Moreover, most prior studies focus on circulating adiponectin protein rather than mRNA expression [16,17], neglecting the upstream regulatory disruptions that may precede clinical manifestations. This study addresses this gap by correlating ADIPOQ gene expression inversely with MDA and IL-6 levels, thus establishing a link between oxidative-inflammatory stress and suppressed metabolic protection at the genetic level in a youthful demographic.

**Strengths and limitations:** one of the key strengths of the study lies in its focus on a younger adult population (aged 18-45 years), which is critical for early identification of individuals at risk for non-alcoholic fatty liver disease (NAFLD) and cardiovascular disease (CVD). Early detection in this age group can aid in timely intervention and prevention strategies, as supported by previous studies emphasizing the importance of early risk stratification in metabolic disorders [10]. The integration of clinical and gene expression data provides a multidimensional approach, enhancing the robustness of the findings and aligning with recommendations for combining molecular and clinical markers in disease risk evaluation.

Furthermore, the use of receiver operating characteristic (ROC) curve analysis to assess the diagnostic performance of biomarkers adds to the analytical strength of the study, offering insights into their predictive utility [18]. Applying Firth logistic regression, a method well-suited for small or sparse datasets, ensures more reliable and unbiased parameter estimates, especially when traditional logistic regression may be prone to bias or non-convergence [19]. These methodological choices contribute to the reliability and scientific validity of the study outcomes.

Just like any other study, the present study has its limitations. As a case-control study, this design limits causal inference and may be prone to selection bias. While adequate for initial comparisons, the sample size restricts subgroup analysis and broader generalizability. Unmeasured confounders like diet, physical activity, and medication use may also influence results. Additionally, biomarkers like MDA and IL-6 are non-specific and can be affected by various conditions, which may limit their diagnostic specificity.

**Interpretation:** in this study, malondialdehyde (MDA) levels were significantly elevated in the case group compared to controls (p < 0.01), indicating increased oxidative stress in individuals with NAFLD and/or cardiovascular risk (Table 1). Our findings support previous research, which consistently linked elevated MDA with enhanced lipid peroxidation in cardiovascular and liver-related metabolic disorders. Huang *et al*. and Seki *et al*. reported similar associations between high MDA levels and disease severity in NAFLD and atherosclerosis, respectively, reinforcing the validity of MDA as a biomarker for oxidative stress in cardiometabolic diseases [20,21].

Interleukin-6 (IL-6) concentrations were also significantly higher in cases (p < 0.01), suggesting an ongoing inflammatory response (Table 1). However, logistic regression analysis showed that IL-6 was not independently associated with case status after adjusting for sociodemographic variables. Thus, while IL-6 levels were elevated, their standalone predictive value for disease classification appears limited. This partially supports existing literature, which has highlighted IL-6 as a marker of systemic inflammation in metabolic and cardiovascular conditions [22], though our data suggest that IL-6 may act more as a secondary marker than a direct diagnostic tool in younger populations.

Adiponectin-encoding gene (ADIPOQ) expression was significantly reduced in individuals with non-alcoholic fatty liver disease (NAFLD) and/or cardiovascular risk compared to healthy controls (p < 0.01), indicating diminished metabolic protection in these individuals (Table 1). This observation is consistent with earlier studies that reported reduced adiponectin levels associated with obesity, insulin resistance, and atherosclerosis. For instance, Jansson *et al*. found that circulating adiponectin levels were significantly decreased in individuals with increased cardiometabolic risk [23]. Similarly, Phillips *et al*. demonstrated a strong positive correlation between plasma adiponectin concentrations and adiponectin mRNA levels (r = 0.80, p < 0.001), with significantly lower gene expression in obese individuals [24]. Furthermore, Altinova *et al*. reported that overweight subjects had significantly lower mean plasma adiponectin concentrations than their normal-weight counterparts (15.0 ± 4.2 ng/mL vs. 17.3 ± 5.6 ng/mL, p < 0.05) [25]. While these previous studies primarily focused on circulating protein levels, our study uniquely contributes to this body of evidence by measuring ADIPOQ expression, suggesting that adiponectin deficiency observed in metabolic diseases may originate upstream at the gene regulation stage. This gene-level insight adds a novel dimension to understanding the pathophysiological mechanisms underlying early-onset metabolic dysfunction in younger adults.

Receiver operating characteristic (ROC) curve analysis revealed that MDA had the highest diagnostic accuracy (AUC = 0.9605), followed by IL-6 (AUC > 0.85), whereas ADIPOQ expression showed poor diagnostic utility (AUC = 0.5) (Figure 1). These findings suggest that while oxidative and inflammatory markers are helpful in distinguishing cases from controls, ADIPOQ gene expression alone may not be a reliable diagnostic marker. This contrasts with prior protein-level studies where adiponectin was reported as a helpful biomarker [17]. Our results highlight a possible discrepancy between gene expression and circulating protein levels.

We observed strong inverse correlations between ADIPOQ expression and both IL-6 and MDA, with more pronounced associations in cases than in controls (Figure 2, Figure 3). These findings suggest that inflammatory and oxidative stress may suppress adiponectin gene expression in individuals with cardiometabolic risk. This supports earlier mechanistic insights, which proposed that pro-inflammatory cytokines and oxidative stress negatively regulate adiponectin production [26,27]. However, the strength and pattern of these associations in a younger demographic, as shown in our study, underscore the early onset of metabolic dysregulation.

The present study highlights the early molecular disruptions involving reduced ADIPOQ expression, elevated oxidative stress (MDA), and inflammation (IL-6) in young adults with NAFLD and/or cardiovascular risk. These alterations reflect underlying pathophysiological processes and may serve as potential early markers or targets for risk stratification and preventive interventions in at-risk young populations.

## Conclusion

This study underscores the pivotal role of oxidative stress, systemic inflammation, and downregulated adiponectin (ADIPOQ) expression in individuals with non-alcoholic fatty liver disease (NAFLD) and/or cardiovascular risk factors, even among young adults. Elevated levels of malondialdehyde (MDA) and interleukin-6 (IL-6) reflect heightened oxidative stress and inflammation, which correlate negatively with ADIPOQ expression, a key regulator of metabolic health. Despite the strong association between these biomarkers and disease states, ADIPOQ expression exhibited limited utility in disease classification, indicating the complexity of these molecular signatures in predicting NAFLD and cardiovascular outcomes. These findings highlight the need for further exploration into the molecular pathways that drive metabolic dysfunction in at-risk youth and emphasize the importance of early intervention to prevent the progression of cardiovascular and hepatic diseases.

### What is known about this topic

- Oxidative stress, inflammation, and reduced adiponectin (ADIPOQ) expression are linked to metabolic disorders like NAFLD and cardiovascular disease.

### What this study adds

- In young adults, elevated MDA and IL-6 levels are associated with lower ADIPOQ expression, though ADIPOQ alone shows limited utility for disease classification, highlighting the complexity of early metabolic risk detection.
